# Cryo-EM analysis of the T3S injectisome reveals the structure of the needle and open secretin

**DOI:** 10.1038/s41467-018-06298-8

**Published:** 2018-09-21

**Authors:** J. Hu, L. J. Worrall, C. Hong, M. Vuckovic, C. E. Atkinson, N. Caveney, Z. Yu, N. C. J. Strynadka

**Affiliations:** 1 Department of Biochemistry and Molecular Biology and the Centre for Blood Research, University of British Columbia, Vancouver, V6T 1Z3 BC Canada; 2 HRMEM facility, University of British Columbia, Vancouver, V6T 1Z3 BC Canada; 3 CryoEM Shared Resources, Janelia Research Campus, Howard Hughes Medical Institute, Ashburn, 20147 VA USA

## Abstract

The bacterial type III secretion system, or injectisome, is a syringe shaped nanomachine essential for the virulence of many disease causing Gram-negative bacteria. At the core of the injectisome structure is the needle complex, a continuous channel formed by the highly oligomerized inner and outer membrane hollow rings and a polymerized helical needle filament which spans through and projects into the infected host cell. Here we present the near-atomic resolution structure of a needle complex from the prototypical *Salmonella* Typhimurium SPI-1 type III secretion system, with local masking protocols allowing for model building and refinement of the major membrane spanning components of the needle complex base in addition to an isolated needle filament. This work provides significant insight into injectisome structure and assembly and importantly captures the molecular basis for substrate induced gating in the giant outer membrane secretin portal family.

## Abstract (web summary)

The bacterial type III secretion system of Gram-negative bacteria uses its core, the needle complex, to penetrate through the infected host cell membrane. Here authors show a near-atomic resolution structure of a needle complex which sheds light on the assembly and function of this nanomachine.

## Introduction

The injectisome is a specialized bacterial organelle that utilizes a type III secretion system (T3SS) to translocate effector proteins from the bacterial cytosol directly into a eukaryotic host. It is found predominantly in pathogenic Gram-negative bacteria and is critical for the virulence of many medically relevant bacteria including the causative agents of plague, typhoid fever, whooping cough, sexually transmitted infections, and major nosocomial infections^1^. Composed of >20 different proteins, the injectisome forms a syringe shaped nanomachine that spans both bacterial membranes and that of the infected host. Forming a continuous passage, it allows for efficient delivery of a variety of effector proteins that in turn serve to affect host cellular targets and functions to the benefit of the pathogen^2^. The major structural unit of the injectisome is the basal body, composed of three highly oligomerized concentric rings spanning the bacterial inner membrane (IM) (PrgH and PrgK in the *Salmonella enterica* serovar Typhimurium SPI-1 T3SS)^3–6^ and outer membrane (OM)^7^ (InvG, a member of the secretin family of OM pores also found in the type II secretion system, type IV pili system, as well as the phage assembly system^8^). Packing within the IM rings of the basal body, the export apparatus consists of five conserved membrane associated proteins (SpaPQRS^9^ and InvA) which are thought to further modulate the T3SS by creating a size restricted inner pore from the cytosol and across the IM^10^. Regulating effector secretion in conjunction with the export apparatus are several soluble components anchored to the cytoplasmic face of the IM and basal body including an ATPase that serves to deliver effector proteins to the injectisome and regulate substrate secretion^11^. Completing assembly, the helical needle filament—composed of early substrates PrgJ (the rod) and PrgI (the needle)—is secreted in a type III secretion (T3S) dependent manner and polymerizes on the periplasmic face of the export apparatus, extending into the cellular milieu^12^. Upon host cell contact, mid substrates are secreted that form a translocon pore (SipB and SipC) in the host cell membrane, establishing a conduit from bacteria to host and allowing for subsequent delivery of late virulence dependent effector proteins.

The injectisome has been the subject of extensive structural analysis from both reductionist and systems standpoints with high-resolution techniques such as X-ray crystallography and NMR used to solve the structures of some of the constituent proteins and traditionally lower resolution methods such as cryo-EM and tomography used to probe the overall architecture^3,6,7,11–18^. Recent advances in single-particle cryo-EM technologies have facilitated a radical improvement in the attainable resolution of the method. We recently published the cryo-EM structure of the prototypical *S*. Typhimurium SPI-1 basal body detailing the molecular architecture of the three oligomeric ring forming proteins that span the bacterial IM and OM in their assembled state, and revealing the massive double walled 60 stranded β-barrel structure of the family of OM secretin portals^7^. In that study, we used a T3S incompetent mutant lacking the N-terminal cytoplasmic domain of PrgH (PrgH_130–392_), which assembled basal body complexes lacking the internal rod/needle filament and with the secretin periplasmic gate in a closed conformation. The atomic details governing the mechanism and specificity of molecular gating in eukaryotic and prokaryotic membrane transport has generated exceptional interest in the past two decades. From smaller ion channels with pore sizes of 3–4 Å through to systems which transport large macromolecular substrates the underlying question is how these systems provide for passage of only select substrates while preventing deleterious release or uptake of other molecules? In the case of the giant secretin OM portals of bacteria, with inner lumens of more than 75 Å^19^, the necessary gating becomes a fascinating and critical functional aspect. To address this and further elucidate the structural basis for injectisome assembly and function, including that of the central needle component, we have conducted here a cryo-EM analysis of the assembled native needle complex resulting in 3D reconstructions of the IM and OM rings at resolutions permitting atomic model building (3.6 Å and 3.9 Å, respectively) and the 3D reconstruction of an isolated helical needle filament at 3.3 Å resolution. Collectively, these structures shed significant light on the process of injectisome assembly, needle architecture and span, and importantly capture the precise molecular details of the open gate conformation of the widely dispersed giant OM secretin portal family.

## Results

### The molecular envelope of the overall needle complex

**Fig. 1:**
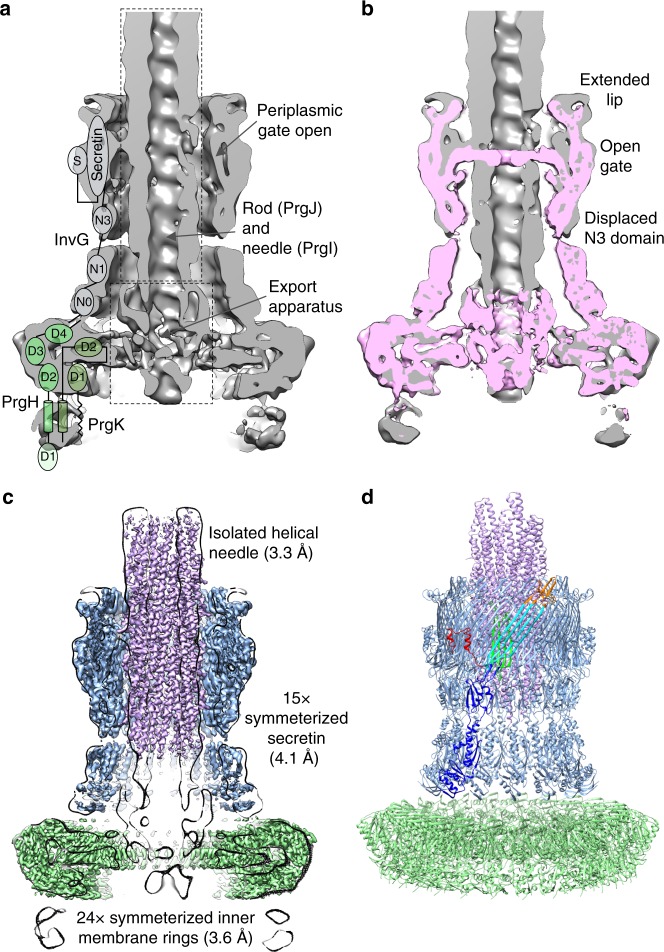
Cryo-EM structures of the injectisome needle complex and isolated needle. **a** Needle complex C1 reconstruction (low pass filtered from 7.4 Å reconstruction to highlight overall features) cut away at the mid-section. The domain annotation of PrgH, PrgK, and InvG is overlaid on the left. Boxed regions indicate the periplasmic region of the export apparatus and the rod/needle filament. **b** Central slice view of needle complex reconstruction (gray) overlaid with the 6.3 Å basal body reconstruction (EMD-8400) (pink). **c** Reconstructions for the 24-fold averaged IM rings (green; 3.6 Å resolution), the 15-fold averaged secretin (blue; 4.1 Å resolution) and the isolated needle (magenta; 3.3 Å resolution). High-resolution reconstructions overlaid on C1 reconstruction shown as central slice (black). The needle was fit into the needle complex C1 map using Chimera and agrees well with the wider part of the needle complex filament. **d** Refined structures for InvG_34–557_ (blue), PrgH_171–364_ (green), PrgK_20–203_ (green), and PrgI_3–80_ (magenta). One monomer encompassing InvG_34–557_ is colored according to structural domains: N0-N3 domains (blue); outer β-sheet (cyan); inner β-sheet (green); secretin domain lip (orange); S domain (red)

We purified intact *S*. Typhimurium SPI-1 needle complexes as previously described^16^. Single particle reconstruction resulted in unsymmeterized maps to 7.4 Å resolution as judged by gold-standard FSC^20^ revealing the molecular envelope for the IM and OM rings of the basal body and the encompassed substructures with attached needle filament (Fig. 1a). Comparison to the previously determined basal body reconstruction^7^ reveals the general localized changes within the core secretin domains necessary to accommodate the assembled needle filament; notably the extended open orientation of the periplasmic gate (Fig. 1b). Outside of these regions, the overall structure is remarkably static between assembly states.

### The structure of the 24-mer IM rings

The 24-fold features of the IM rings in the C1 reconstruction were clearly visible and further C24 symmetry averaging resulted in a reconstruction to 3.6 Å resolution (compared to 4.3 Å for the basal body IM rings; EMD-8398) allowing for refinement of the atomic structures of the periplasmic domains of PrgH_171–364_ and PrgK_20–203_ (Fig. 1c, d, Supplementary Fig. 1, Supplementary Table 1). Consistent with the conserved dimensions of the IM rings between the needle complex here and that of the previous basal body reconstructions (Fig. 1b), the refined PrgH and PrgK ring atomic models determined from each overlap closely (overall Cα RMSD 0.7 Å) suggesting an inherently highly stable structure with no significant conformational change required in this region upon needle assembly.

### The export apparatus and needle filament

**Fig. 2:**
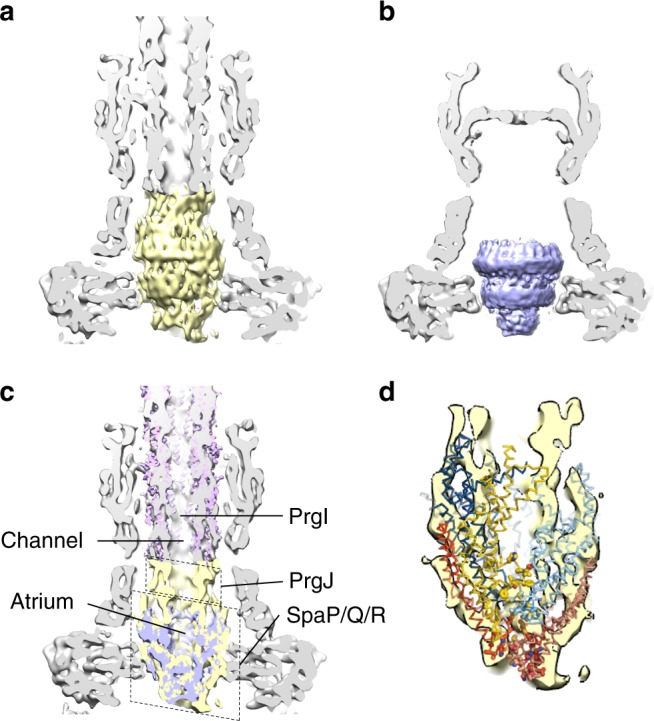
The export apparatus forms an asymmetric substructure composed of SpaP and SpaQ and SpaR. **a** The region of the needle complex C1 reconstruction corresponding to the export apparatus and putative rod colored yellow. Slabbed remainder of reconstruction colored gray. **b** The region of previous C1 basal body reconstruction^7^ corresponding to the export apparatus colored purple. The map features a flat, symmetrical pore, averaged out by the signal of the 24-mer IM rings (24-mer repeating features evident on upper surface). Slabbed remainder of reconstruction colored gray. **c** Slabbed view of overlay of **a** and **b** showing export apparatus region from needle complex map (yellow) is asymmetric compared to the same region from the basal body map (purple) (lower box). The 3.3 Å needle map (magenta) fits well (correlation coefficient = 0.95) into the wider part of the filament density. We propose the boxed narrower region between the base of the filament and the export apparatus represents the PrgJ rod, which has been proposed to form a short adaptor between export apparatus and needle^22^. **d** The recent structure of the *Salmonella* flagellar export apparatus^23^ FliP (blue)/Q (red)/R (yellow)) forms a complex with stoichiometry of 5:4:1 and overlays very well with the needle complex export apparatus substructure. The subunits form a helical assembly ideal as a structural foundation for the assembly of the rod and needle filament. Atomic model figure reproduced under the CC-BY 4.0 International license

The low-resolution substructure density localized within the central lumen of the stacked IM PrgH and PrgK rings at the base of the needle filament has historically been referred to as the socket and cup. It is present in native and T3S incompetent mutants alike^5,7^, the latter of which consequently lack the rod/needle filament (themselves T3S substrates). Mutational, mass spectrometry and low resolution EM analysis have attributed this substructure density to regions of the IM associated export apparatus components SpaP, SpaQ, SpaR, and SpaS^9,15,21^, with knockout of one or more removing the substructure in vitro and in situ^9,13^. Remarkably, a recent cryo-EM structure of the isolated recombinant export apparatus complex from the bacterial flagellar system (long recognized to encode several analogous IM components to the injectisome) shows that this substructure is composed of the flagellar orthologues FliP/Q/R (25–30% sequence identity with *Salmonella* SPI-1 SpaP/Q/R) in their entirety despite predictions they were integral membrane proteins with 4, 2, and 6 predicted transmembrane domains, respectively. The complex adopts a helical assembly of dimensions ~90 × ~60 × ~60 Å with 5:4:1 stoichiometry (FliP/Q/R) with the single FliR bridging the IM facing FliQ tetramer and the more periplasmic facing FliP pentamer and elegantly explains previous cross-linking and mass spectrometry studies^9,21–23^. Complicated by the local symmetry mismatch (e.g., with the 24-mer IM rings which encompass the export apparatus) and potential relative conformational variation, we have been unable to resolve the high resolution structural features of this substructure in the current needle complex data or in the prior basal body reconstruction. However, whereas the latter presented a rather flat, symmetrical pore due to averaging out of the local structure (see Fig. 2b), the needle complex reconstruction here provides an inherently more detailed, asymmetric substructure than previously observed (Fig. 2a–c). Overlay of the flagellar FliP/Q/R structure matches well throughout suggesting the structure of the flagellar and non-flagellar T3SS export apparatus will be conserved, providing evidence for the precise span of the export apparatus within the assembled T3SS (Fig. 2d).

**Fig. 3:**
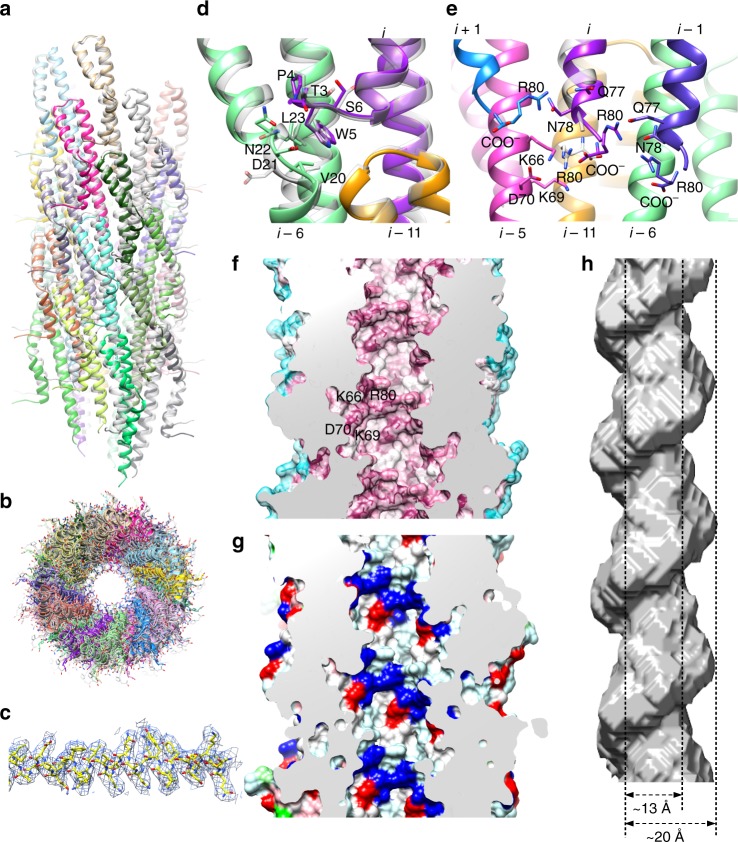
Structure of the PrgI needle filament at 3.3 Å defines a spiral groove for effector secretion. **a**, **b** Side and top views of the helical packing of the PrgI needle with 5.7 monomers per turn and a helical rise of 4.33 Å. The ssNMR structure of PrgI (PDB 2LPZ; gray transparent) is overlaid showing the subtle differences in helical parameters create a small but accumulating change in subunit packing. Side chains, resolved in the reconstruction here, are shown in **b** highlighting the constriction of the interior channel. **c** Representative density (3.3 Å resolution). **d** Zoomed in view showing interface of *i, i*−6, and *i*−11 monomers formed around the N-terminal loop of *i*. The ssNMR structure of PrgI (gray transparent) is overlaid. The variable N-terminal loop here is packed closer to the kinked helix of monomer *i*−6. The kink (residues 20–23) adopts a different conformation compared to the ssNMR structure. **e** Zoomed view of the interaction network around the C-terminal Arg_80_. The ssNMR structure is overlaid on monomer *i* showing the Arg_80_ side chain and carboxylate in a flipped orientation. Here, the Arg_80_ side chain guanidinium group interacts with Gln_77_ and Asn_78_ on monomer *i*–1 while the carboxylate interacts with Lys_66_ on *i*–5. **f** The interior channel is significantly more conserved than the outer surface (conservation colored from cyan (low) to maroon (high)). The cluster of conserved residues around the C-terminus as in **e** define the raised edge of a right handed spiral that extends the length of the lumen. The groove is lined by repeating deeper pockets defining the path of effector secretion. **g** Same view as in **f** colored according to residue type: hydrophobic–gray, aromatic–light pink, polar–light cyan, positive–blue, negative–red, cysteine–light yellow, proline–light green, glycine–green. The raised edge of the groove is demarcated by charged residues while the groove itself is predominantly polar and hydrophobic. **h** Surface corresponding to the interior needle lumen highlighting the right handed spiral and dimensions of the channel

The needle filament observed in our reconstruction forms a continuation of the export apparatus with the internal channel diameter the same as the export apparatus atrium (Fig. 1b, Fig. 2c). The needle (PrgI) is proposed to be initiated by a homologous protein historically termed the rod (PrgJ)—both detected by MS analysis here (Supplementary Table 2) but absent, as designed, from the purified secretion incompetent basal body^7^. Although the general dimensions and span of the filament are clearly observed, like with the export apparatus and the issues with localized symmetry mismatch with surrounding components, we were unable to sufficiently resolve the high-resolution structural features within the assembled needle complex to allow model building and refinement. To further study the atomic details of the needle therefore, we isolated native filaments from assembled needle complexes and subjected them to cryo-EM analysis resulting in a reconstruction with helical averaging to 3.3 Å resolution with excellent density for side chains throughout (Supplementary Fig. 2, Fig. 3c, Supplementary Table 1) allowing us to build a de novo model of PrgI lacking only the first two N-terminal residues (Figs. 1c–d, 3). The PrgI monomer adopts a helix-turn-helix motif that polymerizes via an extensive network of interactions along both helices with surrounding subunits (Fig. 3a, b). The structure supports the solid-state NMR model of PrgI presented by Loquet et al.^18^ (Cα RMSD 1.5 Å over 78 residues), which was fundamentally different from prior low resolution cryo-EM derived models^24,25^; however, some notable differences in the monomeric structure and helical packing, as well as the definition of the majority of side chain positions extend our understanding of the intra-subunit interactions and the internal channel passaged by effectors. The kinked loop in the N-terminal helix (residues 20–23) is more extended and forms a tighter interface with the N-terminus of monomer *i* *+* 6 while the C-terminal Arg_80_ has its side chain and carboxylate flipped, altering the interactions with monomers *i* −1, *i* –5, and *i* – 6 (Fig. 3d, e). The refined polymer forms a helical assembly with approximately 11 subunits per two turns but a minor difference in the helical rise compared to the solid-state NMR model (helical rise 4.33 Å vs. ~4.2 Å, respectively) creates a small but significantly accumulating shift in subunit packing (Fig. 3a, b). Along with the specific structural changes at the C-terminus, this creates a right handed spiraled groove with polar and hydrophobic character extending along the needle lumen which was not observed in the NMR model, with a cluster of conserved residues around the C-terminus defining the raised edges of the groove (Fig. 3f–h). Taking into account the resolved side-chains here (Fig. 3b, c), the axial diameter is considerably less than previously measured in lower resolution reconstructions^24,26,27^ (~15 Å vs. ~25 Å, only very closely accommodating a single helix for example) suggesting unfolded or partially unfolded secreted effectors would necessarily track along the deeper and longer helical path during passage (Fig. 3f–h). Indeed, previous scanning alanine mutations in the *Shigella* orthologue MxiH showed that mutation of conserved charged residues that here define the raised groove (equivalent to PrgI Lys_66_, Lys_69_, Asp_70_, Arg_80_; Fig. 3e–g) had differential effects on needle polymerization and length, secretion, and regulation^28^ suggesting a specific contribution of the interior needle channel surface in the hierarchical secretion of T3S effectors as opposed to merely providing a channel for passive diffusion. The helical nature of the secretion channel also perhaps raises the intriguing possibility that an as yet undefined rotational force, perhaps linked to energy from the proton motive force required for secretion^29,30^, might contribute to effector secretion. Fitting of the cryo-EM derived model into the C1 needle complex map shows a good agreement (Chimera correlation coefficient = 0.95) between the wider part of the filament density (Figs. 1c, 2c). This needle placement suggested by our data here, anchored deep into the secretin lumen in keeping with its needed stability within the relatively severe mechanical environment of the infected cell, is consistent with a recently proposed role of the protein PrgJ as a relatively small bridge or adaptor between the IM export apparatus and the needle filament^22^ rather than an extended rod as its historical name unfortunately had predicted (here accounting for the narrowing region of density at the base of the filament (Fig. 2c)). Taken together, these structures now demonstrate that the SpaP/Q/R export apparatus complex is positioned to provide a structural foundation for the assembly of the needle filament with the helical, pseudo-hexameric arrangement of the central 5× SpaP and 1× SpaR subunits an ideal template for the downstream T3S dependent polymerization of the helical filament, with several (likely hexameric) copies of PrgJ (predicted stoichiometry 3–6 by mass spectrometric analysis^22^) putatively assembling onto the SpaR/P platform (cross-linking demonstrates close interaction^21^) and in turn providing a helical template to initiate the helical needle polymerization.

### The open state of the OM secretin gate

**Fig. 4:**
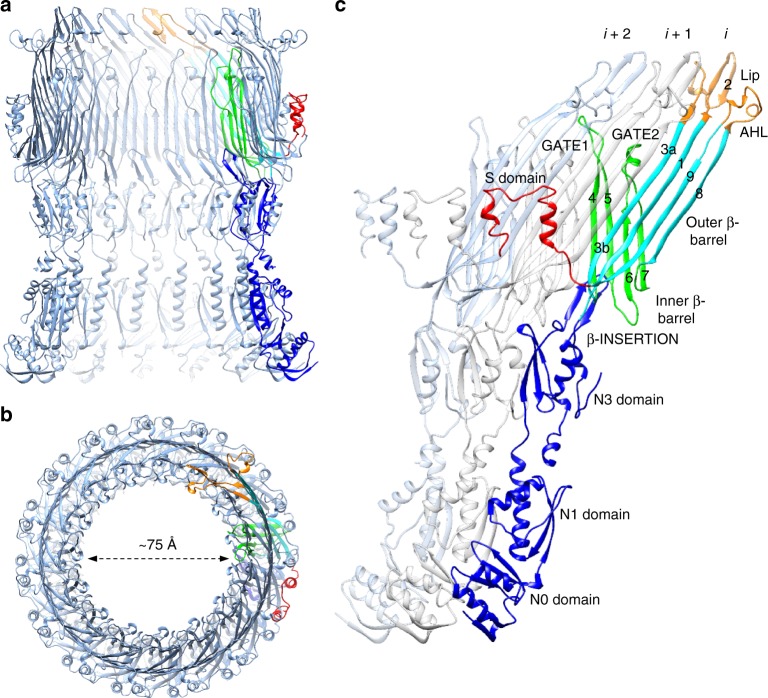
Structure of the InvG secretin pore in the open state. InvG_34–557_ secretin pore structure viewed from (**a**) side slab, (**b**) top (OM perspective) and (**c**) three monomers highlighting inter-domain and intra-domain packing of monomers *i*, *i* + 1 and *i* + 2. One monomer colored according to structural features as per Fig. 1. Secretin domain β-strand numbers as per our previous closed InvG structure^7^ indicated in **c**

In these needle complex samples, the strong 24-mer symmetry of the IM rings dominates the 2D class averages, a consequence of which is that the region of the C1 needle complex reconstruction corresponding to the ~1 MDa OM secretin component is somewhat smeared out and lacked obvious symmetric or high-resolution features. We therefore employed a partial signal subtraction and masked focussed local refinement procedure^31^ with the obvious 15-fold symmetry resulting used subsequently in averaging to produce reconstructions to 4.1 Å resolution for the entire secretin and 3.9 Å resolution for the core secretin structure encompassing the N3, secretin and S domains (Fig. 1c, d, Supplementary Fig. 3). The density in the core region (InvG_176–557_) showed defined secondary structure with the majority of side chains clearly visible (Supplementary Fig. 3h) allowing for unambiguous building and refinement of the structure including significant additionally resolved and structurally changed regions from the previously determined closed form^7^. Although the more peripheral N-terminal N0 and N1 domains were of lower local resolution (Supplementary Fig. 3g), the map was of sufficient quality to reveal secondary structure elements for the N1 domain in particular allowing us to position 15 copies of our prior InvG_34–173_ crystal structure for that domain (PDB 4G08^5^) (Supplementary Fig. 5e) further expanding the defined model of the injectisome. The final secretin model spanning all domains (residues 34–557, N3 domain loop 228–251 disordered; Fig. 4, Supplementary Table 1) was refined with density restrained symmetry refinement in Rosetta and notably reveals the dramatic conformational changes involved in secretin gate opening during needle assembly, a fundamental and functionally critical aspect of this giant gated pore family.

**Fig. 5:**
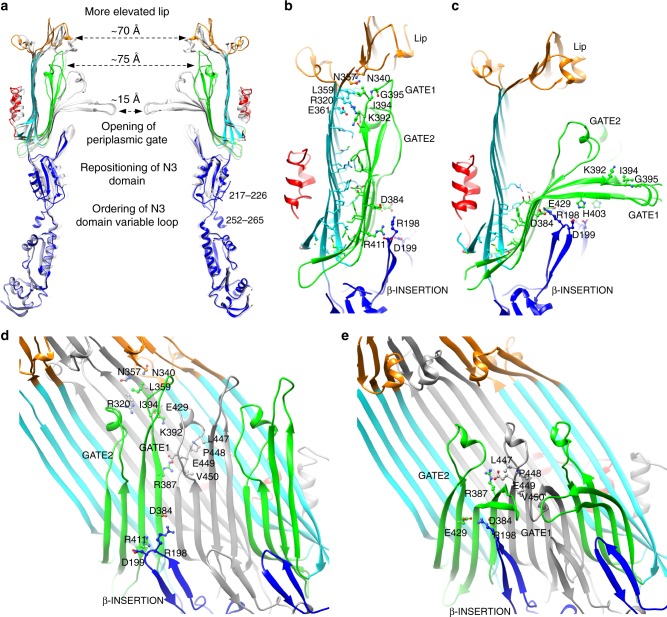
Structural changes involved in InvG gate opening. **a** Comparison of the InvG secretin gate open state (colored as per Fig. 1) and the closed state (gray transparent). The major structural changes are the ordering of the N3 domain variable loop (residues 217–226 and 252–265, disordered in other secretin structures) and accompanying change in N3 domain position, the opening of the periplasmic gate involving the repositioning of GATE1 and GATE2, and the more elevated lip region caused by interactions with GATE1 and the insertion of the assembled needle filament. **b** and **c**–side view–and **d** and **e** inside view–compare the interface between outer and inner β-barrels in the open and closed states, respectively with accompanying interface areas of 2111 Å^2^ and 1153 Å^2^, residues forming the open and closed interfaces shown as sticks in **b** and **c**. The core interface at the base of the β-sandwich formed by the outer and inner β-barrels is predominantly hydrophobic while the region formed by the extension of the GATE1 and GATE2 hairpins is mostly polar in nature. Key interactions defining the open and closed conformations between the N3 domain β-INSERTION, the inner β-barrel GATE1 and GATE2 motifs, and the upper outer β-barrel and lip are labeled and shown as ball and stick. The middle subunit is colored gray in **d** and **e** to define the outer and inner β-barrels. The closed gate conformation is supported by interactions of the N3 β-INSERTION, specifically Arg_198_, with the kinked regions of the GATE1 (Asp_384_) and GATE2 (Glu_429_) hairpins and further supported by the surrounding network of interactions. In the open gate conformation, the N3 β-INSERTION interface is disrupted and the gate forming GATE1 hairpin is now extended toward the lip and packed against the outer β-barrel with Ile_394_ at the tip packing in a hydrophobic notch formed by the side chains of Arg_320_, Asn_340_, Asn_357_, and Leu_359_. The GATE2 hairpin undergoes a significant rotation with residues stabilizing the closed gate—Leu_447_, Pro_448_, Glu_449_, and Val_450_—packing against the outer β-barrel wall. A salt bridge between GATE2 Glu_449_ and GATE1 Arg_387_ is maintained between closed and open conformations

Overlay of the needle complex and prior closed basal body reconstructions illustrates that the primary sites of conformational change upon needle assembly are in the conserved core secretin region: the N3 ring domain—the tightly oligomerized foundation upon which the secretin assembles^7^—and the inner β-barrel and membrane associating lip of the central secretin domain, all of which have reoriented to accommodate the presence of the needle filament (Fig. 1b). The N-terminal N0 and N1 domains, outer β-barrel and C-terminal surface exposed S domain conversely show very little structural difference between assembly states; as with the static IM ring components they appear to provide a highly stable scaffold or shell to buttress the inner conformational changes required for needle passage. The most dramatic conformational change upon needle assembly is in the inner β-barrel. In the previously observed closed state^7^, the extended β-hairpin formed by strands 4 and 5 (numbering consistent with the closed InvG structure^7^, here termed GATE1 based on mutational analysis in secretin pIV^32^) is kinked and twisted around residues Asn_386_ and conserved Gly_407_, with the spoke like radial projections afforded by the pentadecameric symmetry forming a collective barrier across the ~75 Å secretin lumen that is permeable only to small molecules^32–34^. In the T2SS, mutation of the Asn_386_ equivalent in PulD (Gly_458_) increased antibiotic sensitivity^35^ and accordingly resulted in a partial opening of the gate in the cryo-EM structure of GspD (Gly_453_)^36^. In the fully open state defined here, the GATE1 hairpin is now unkinked, with the residues at the tip having moved position by a remarkable ~40 Å (measured from the Gly_395_ Cα) (Fig. 5). The open position is stabilized by intimate packing against the outer β-barrel of itself and neighboring monomer *i* + 1 (monomers defined clockwise looking down from the OM), and extending vertically toward the lip region of monomer *i* + 2 (Figs. 4c, 5b–d) with a dramatic increase in buried surface area between the inner and outer β-barrels (2111 Å^2^ per monomer compared to 1153 Å^2^ in the closed state) (Fig. 5b–e). Side chain density is defined for the large majority of residues along the length of both GATE1 strands with the exception of 5 residues (Glu_396_-Ala_400_) at the tip. Although no longer kinked, the twist in GATE1 is maintained in the extended state such that strand 4 is oriented closer to the outer β-barrel forming multiple specific interactions. Of note, Lys_392_ and Ile_394_ at the C-terminus interact with residues on the outer β-barrel and lip of monomers *i* + 1 and *i* + 2 (Fig. 5b–d). In our previous secretion assays, mutation of either Lys_392_ or Ile_394_, as well as Gly_395_, which forms a tight turn in both closed and open gate conformations, significantly impacted secretion^7^. The inner β-barrel strands 6 and 7 (termed GATE2 here) are straightened around Gly_430_ and Gly_451_ compared to the closed form. More significantly, the GATE2 loop (residues Asp_433_-Gly_451_) undergoes a 180° rotation, effectively repositioning the regions oriented above the GATE1 hairpin in the closed state against the outer β-barrel in the open state (Fig. 5). These changes in GATE1 and GATE2 of the inner β-barrel result in an increase in the secretin pore diameter from ~15 Å in the closed state to a remarkable ~75 Å in the open state (Figs. 4b, 5a).

The secretin outermost lip constitutes a kinked 45-stranded β-barrel, capping the top of the 60-stranded outer β-barrel wall^7^ (Fig. 4). With one strand less per monomer, the well-ordered lip was observed in the basal body closed reconstruction to collapse inwards to structurally accommodate the reduced strand count. Decorated on the exterior by an amphipathic helical loop (the AHL)—the most conserved sequence across the secretin family^7^—this region was proposed on structural features to be central to membrane association, BAM-independent insertion and OM span^7^. In the needle assembled open state here, the lip has been pushed up and out to accommodate the passage of the filament, increasing the outer pore diameter from ~65 Å in the closed state to ~70 Å in the open state (Fig. 5a). The presence of the needle supports a more upright lip orientation consistent with the recent in situ cryo-electron tomography structure of the *S*. Typhimurium T3SS which demonstrated the membrane invagination we had noted previously^13,37,38^ to be more pronounced in a mutant lacking the needle compared to the assembled needle complex^13^ (Supplementary Fig. 4). Interestingly, recent structures of several T2SS secretins have revealed their lip region consists of 4 rather than 3 β-strands per monomer and as such present a non-kinked continuation of the outer β-barrel wall^36,39^ (Supplementary Fig. 5a, b). Sequence analysis suggests the three-stranded lip architecture observed for InvG will be common in other T3SS secretins and may reflect the necessity for a closer fitting and more stable interface with the needle, which is more statically attached in the injectisome than, by comparison, the dynamically attached (pseudo)pilus of the T2SS or T4PS which are in a fluctuating state of extension and retraction.

The N3 domain abuts the periplasmic base of the inner β-barrel formed by GATE1 and GATE2 (Figs. 4, 5) and has been demonstrated through mutagenesis to be essential for secretin oligomerization^35,40,41^. N3 belongs to the family of small mixed α/β domains we previously termed ring building motifs^4^ (RBMs) which have been observed in numerous ring forming components from different secretion systems. Although often sharing a common general oligomerization interface^4,5,32,33^ the variation of non-covalent interactions and surface areas observed suggest inherent degrees of stability in RBM oligomerization that may reflect their role in assembly. The structure of InvG^7^ and subsequent T2SS secretins^36,39^ demonstrated that the N3 domain forms a ring oligomer with a consistently hydrophobic RBM interface that is also intimately associated with the underside of the inner β-barrel of the neighboring *i* + 1 monomer (Fig. 4c, Supplementary Fig. 5a, b), providing a stable foundation for secretin oligomerization. Here, in the open state, the N3 domain is rotated outwards slightly (Fig. 5a). The β-hairpin insertion specific to T3SS secretin orthologues (referred to here as β-INSERTION), observed to form a strut supporting the conformation of GATE1 and GATE2 in the closed state^7^, is pulled away and twisted, disrupting the specific interactions of conserved Arg_198_ at the β-INSERTION turn with Asp_384_ and Glu_429_ at the kinked regions of GATE1 and GATE2, respectively (Fig. 5b–e). The neighboring conserved Asp_199_ also shifts to interact with Arg_411_ at the base of GATE1 in the open state (Fig. 5b–e). Mutation of Asp_199_ completely abrogated secretion with Arg_198_ having a milder phenotype in our previous assays^7^ supporting the importance of these observed interactions in the two functional open/closed conformational states of InvG.

The quality of the needle complex local reconstruction in the OM secretin region has also permitted the observation of further structural features previously not resolved: First, we have been able to model additional sequences of the variable insertion (residues 217–267) between the first helix and second strand of the N3 domain. The now resolved 217–227 and 252–267 residues demonstrate an unexpected contribution to the structural stabilization of the secretin N domains (Fig. 5a, Supplementary Fig. 5c, d). In the prior basal body reconstruction, we suggested this unresolved loop might account for the map features forming a periplasmic constriction evident at lower contour levels^7^. However, with the needle filament occupying the secretin lumen in the assembled state here, the now clear atomic details of the loop show it threads out between the N3 and N1 domains and participates in both intra and inter domain interactions. The N-terminal region (residues 217–227) contributes to the N3 domain RBM interface, effectively chelating between the first strand of monomer *i*–1 and the second helix of monomer *i* (Supplementary Fig. 5c,d) while residues Met_252_-Gln_260_ form an α-helix which packs between the N1-N3 domain loops of monomer *i* and *i*−1 and interacts with the N1 domain of monomer *i* (Supplementary Fig. 5c,d). Collectively these additional interfaces serve to further anchor and stabilize the needle bound form of the injectisome at the point of greatest structural change. Second, we have been able to position the peripheral N0 and N1 domains, which bridge the periplasmic space to the IM rings with the N-terminal residues of the N0 domain interacting with the C-terminus of PrgH^7,12,17^ (Fig. 1a, Supplementary Fig. 5e; see above). Interestingly, while the structure validates our previous Rosetta density guided symmetry models of the InvG_34–172_ crystal structure^5,7^, the data here confirms the direction of the N1 domain RBM interface is indeed opposite to the N3 domain (Supplementary Fig. 5a). This is in contrast to the recent cryo-EM structures of several T2SS secretins^36,39^ where the N1 and N2 (a distinct RBM motif not present in T3SS secretins) domains are shown to have the same orientation with the relative inter N domain packing more staggered compared to InvG (Supplementary Fig. 5a, b). These rather unexpected distinctions again clearly underlie the plasticity of the RBM in modulating periplasmic span and inner membrane coupling in these functionally diverse secretion systems.

### A mechanism for substrate mediated secretin gate opening

**Fig. 6:**
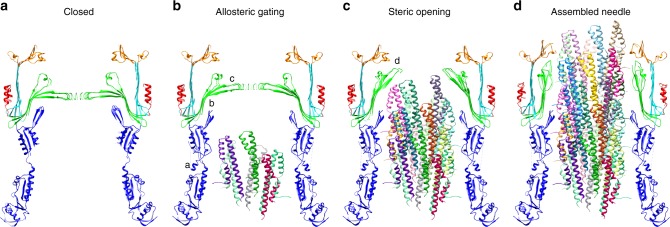
Proposed secretin gating mechanism. **a** Closed secretin. **b** Initial rod/needle polymerization within the secretin lumen contacts the N3 domain triggering the ordering of the variable N3 loop^a^ providing lateral stability and altering the N3 domain-inner β-barrel interface^b^. This in turn disrupts the interactions of the N3 β-INSERTION with the GATE1 and GATE2 kinks and unlocks the gate^c^. **c** Continued needle polymerization sterically pushes the gate open^d^. **d** Final assembled needle with fully open gate

We previously envisaged a substrate induced gate opening mechanism for the OM secretin during needle assembly based on comparison of the basal body structure to previous low resolution (10–20 Å) studies of the needle complex^17,42^. The near-atomic resolution structure of the needle complex here provides details of the discrete structural changes occurring at the molecular level and permits further elaboration. We hypothesize a gate opening mechanism broadly divided into two stages: an initial allosteric transition to unlock the gate and a secondary steric phase to push the gate open. Rod/needle polymerization within the secretin lumen would bring the filament into contact with the N3 domain, including the regions of the N3 domain loop that account for the periplasmic constriction visible in the isolated cryo-EM basal body^7^ and in situ cryo-ET reconstruction^13^, triggering a reorientation/ordering to adopt the conformation we observe here and contributing lateral stability to withstand the continued force of the assembling needle (Fig. 6a, b). The accompanying change in N3 domain orientation we observe alters the interface with the inner β-barrel, which we propose triggers an allosteric transition that effectively unlocks the gate (Fig. 6b). Central to this appears to be the disruption of interactions of the β-INSERTION with GATE1 and GATE2: Arg_198_ at the β-INSERTION turn is at the center of a network of polar and hydrophobic interactions that serve to stabilize the closed gate conformation of GATE1 and GATE2 (Fig. 5b–e). The reorientation of β-INSERTION and disruption of this interface could enable the downstream conformational changes associated with gate opening. Supporting an important functional role, mutation of Arg_198_ and neighboring Asp_199_ impacted secretion in our previous assays^7^. GATE2, although not contributing directly to the physical barrier per se (the role of GATE1), appears to play rather a critical buttressing role for GATE1, undergoing a significant displacement between closed and open states (Fig. 5b–e). In the closed state, residues at the N-terminus of strand 7 in GATE2 pack between kinked GATE1 β-hairpins of monomers *i* and *i*–1 holding them in a closed position (Fig. 5e), whereas in the open state this same region is rotated 180° and packed against the outer β-barrel and the extended GATE1 of monomer *i* (Fig. 5d). A specific interaction between GATE2 Glu_449_ and Arg_387_ of GATE1 of monomer *i*–1 is maintained between the two states suggesting the movement of these two hairpins occurs in concert (Fig. 5d, e), with the rotation of GATE2 potentially initiating the lifting of the GATE1 and/or providing structural support to guide the extended β-strands during opening. Mutants found to increase the permeability of the filamentous phage secretin pIV map to GATE2 in addition to GATE1 (Supplementary Fig. 6) highlighting the influence its conformation has on the extent of gate opening. Continued needle polymerization within the secretin lumen could provide additional energy to sterically push the gate open with the resulting reorientation of the entire inner β-barrel we observe (Fig. 6c, d). The resulting complementary fit, notably at the T3SS specific adaptations of the N3 domain β-INSERTION and three-stranded collapsed β-barrel outer lip, and complementary electrostatics of the N3 and β-INSERTION (Supplementary Fig. 7) would contribute to the stable anchoring of the needle through the OM, essential for the assembled needle complex that must endure considerable extracellular forces in the infected host.

## Discussion

In this work, we have solved the cryo-EM structures of the T3SS needle complex and isolated needle filament at resolutions permitting atomic modeling. These structures provide essential understanding of injectisome structure and importantly provide the view and molecular understanding of the pivotal elements involved in substrate specific gating across one of the largest known membrane spanning portals. This work and the resolution it affords in turn paves the way for potential structure-based design of antimicrobials (compounds that block gate opening or needle polymerization for example) against the many important clinical and community pathogens that rely on a T3SS for their virulent effects.

## Methods

### Expression and purification

The needle complex was purified as follows with similar methods as previous work^16^. The ΔflhD knockout *Salmonella* strain SL1344^43,44^was transformed with a plasmid expressing the T3SS transcription activator HilA on a pBAD plasmid. The cells were grown in LB with addition of 0.3 M NaCl with low aeration at 37 °C to OD_600_ 0.6. Then 0.12% L-arabinose was added to induce expression until the early stationary phase. Cells were harvested by low speed centrifugation and lysed by adding 0.35% lauryldimethylamine oxide (LDAO) in lysis buffer (150 mM phosphate pH 7.4, 0.5 M sucrose, 1.4 mg/ml lysozyme, 12.2 mM EDTA). The needle complex was obtained by centrifugation at 230,000 × *g* using a 45 Ti rotor (Beckman) for 45 mins and resuspended in buffer A (10 mM phosphate pH 7.4, 0.5 M NaCl, 0.5% LDAO), before loading on to a 27.5% CsCl gradient for overnight centrifugation. All the fractions from the gradients were examined on SDS PAGE to determine which fractions contained the needle complex. The fractions that contained the needle complex were further purified on Superose 6 10/300 GL column (GE healthcare) with buffer B (10 mM phosphate pH 7.4, 0.5 M NaCl, 0.1% LDAO).

Isolated needles were purified as follows: non-flagellated ΔPrgI *Salmonella* Typhimurium was complimented with PrgI and hilA on pBAD plasmid. The cells were grown under the same conditions as above. The filaments were removed by vigorously stirring. The intact cells were removed by low speed centrifugation. Macromolecular contaminations were removed with a 45,000 × *g* spin using a 45 Ti rotor and needle filaments were collected with a 120,000 × *g* spin for 1 h. The pellets were resuspended in TE buffer and loaded on 38.5% CsCl gradient. The fraction that contained needle filaments were used for Cryo-EM study.

### Cryo-electron microscopy

For the needle complex, a thin carbon layer was floated onto C-flat™ Holey Carbon (1.2/1.3) grids. Aliquots of 3 μl of the needle complex sample were applied to glow-discharged grids (20 s on carbon side). The grids were blotted for 3 s at 100% humidity with an offset of −20 and plunge-frozen into liquid ethane using a Vitrobot Mark IV (Thermo Fisher, formerly FEI Company). Grids were imaged on a 300 keV Titan Krios cryo-electron microscope (Thermo Fisher, formerly FEI Company) equipped with a Falcon 3 direct electron detector. Images were taken on the Falcon 3 in electron counting mode at a calibrated magnification of ×47,000, corresponding to 1.75 Å per physical pixel. The dose rate on the specimen was set to be 0.6 electrons per Å^2^ per s and total exposure time was 60 s, resulting in a total dose of 40 electrons per Å^2^. With dose fractionation set at 1.5 s per frame, each movie series contained 38 frames and each frame received a dose of 1.05 electrons per Å^2^. Fully automated data collection was carried out using EPU software with a nominal defocus range set from −1.25 to −4 μm^45^.

For the isolated needle, aliquots of 2.5 μl of the needle filament sample were applied to glow-discharged (60 s on carbon side) Quantifoil grids (Copper, 300 mesh, R1.2/1.3). The grids were blotted for 3 s at 100% humidity and plunge-frozen into liquid ethane using a Vitrobot Mark IV (Thermo Fisher, formerly FEI Company). Grids were imaged on a 300 keV Titan Krios cryo-electron microscope (Thermo Fisher, formerly FEI Company) equipped with a spherical aberration corrector, an energy filter (Gatan GIF Quantum) and a post-GIF Gatan K2 Summit direct electron detector. Images were taken on the K2 camera in dose-fractionation mode at a calibrated magnification of ×37,037, corresponding to 1.35 Å per physical pixel (0.675 Å per super-resolution pixel). The dose rate on the specimen was set to be 5.5 electrons per Å^2^ per s and total exposure time was 10 s, resulting in a total dose of 55 electrons per Å^2^. With dose fractionation set at 0.25 s per frame, each movie series contained 40 frames and each frame received a dose of 1.37 electrons per Å^2^. An energy slit with a width of 20 eV was used during data collection. Fully automated data collection was carried out using SerialEM with a nominal defocus range set from −1.2 to −2.5 μm^46^.

### Image processing

For the needle complex, a total of 1110 movie series were collected at 1.75 Å per pixel. Motion correction was done using MotionCor2^47^. The contrast transfer functions (CTFs) of the summed and dose weighted micrographs were determined using CTFFIND4^48^. Approximately 2000 particles were manually boxed out from selected micrographs to generate reference-free 2D-class averages by Relion^49^. The representative 2D-class averages were also used as templates for automated particle picking for the entire dataset in Relion. With 88,687 particles automatically picked, reference-free 2D classification was performed in Relion and 82,678 particles were kept in the good class averages. 3D classification in Relion generated one good 3D map out of four with 58,198 particles. 3D refinement was performed with these particles using Relion, without or with imposing 24-fold symmetry resulting in reconstructions to 9.4 Å and 4.6 Å, respectively based on Fourier shell correlations (FSCs)^20^ calculated from unmasked maps in Relion at 0.143 criterion without post-processing. After post-processing FSCs calculated from masked maps in Relion at 0.143 criterion is 7.4 Å and 3.6 Å, respectively. Local resolution estimations were performed using Relion with the unmasked C24 map, showing that certain regions exhibit better resolvability than the overall resolution. To resolve the secretin region, particle subtraction and masked focus alignment refinement were used^31^. The non-secretin region was subtracted from the rest of the micrograph in Relion and the signal subtracted particles were subjected to reference free 2D-class averaging by Relion^49^. 3D classification in Relion with 15-fold symmetry generated one good 3D map out of four with 26,280 particles. 3D refinement with 15-fold symmetry results in the secretin region resolved at 4.4 Å based on FSCs calculated from unmasked maps in Relion at 0.143 criterion without post-processing and 4.1 Å after post-processing. Local resolution estimations were performed using relion with the unmasked C15 map, showing that certain regions exhibit better resolvability than the overall resolution. The secretin and N3 domain was processed in same way with 34,096 particles and the final resolution after post-processing was 3.9 Å.

For the isolated needle, a total of 3753 movie series were collected at super resolution (0.675 Å per pixel). Motion correction was done using Unblur^50^, with data binned by two (1.35 Å per pixel after binning), and all 40 frames were aligned and summed to a single micrograph with dose-filtering using Sum_movie for further processing^50^. The contrast transfer functions (CTFs) of the summed micrographs were determined using CTFFIND4^48^. Helical processing the helical filament was done following the procedures in Relion^49,51^. Manual picking of the helical filament was done for 30 micrographs and ~6000 helical segments were extracted that generated initial 2D-class averages as templates for automated particle picking for the entire dataset. With ~1,220,000 particles automatically picked, reference-free 2D classification was performed and ~867,000 particles were kept in the good class averages. Initial helical symmetry parameters were known and used from earlier work^24^. 3D classification with local searches of helical symmetry generated four classes with variations in helical symmetries. Further high-resolution classifications and auto refinements were performed in each helical group and generated final maps after semi-automated post-processing. The final map of Group 1 (41,700 particles included) was solved at 6.8 Å resolution with helical rise of 4.45 Å and turn 63.34°. The final map of Group 2/4 (combined initial Class 2 and 4, 53,000 particles included) was solved at 3.3 Å resolution with helical rise of 4.33 Å and turn 63.34°. The final map of Group 3 (16,500 particles included) was solved at 7.2 Å resolution with helical rise of 4.21 Å and turn 63.44°. All resolutions were determined at a criteria of 0.143 FSC using gold-standard refinement procedures and high-resolution noise substitution to correct soft-mask effects^20^.

### Model building and refinement

For the IM rings, the cryo-EM basal body atomic structure of the PrgH and PrgK rings (PDB 5TCP) was docked into the 24-fold averaged needle complex reconstruction with Chimera^52^ and refined using both Phenix real space refine^53^ and density-guided iterative local refinement as implemented in Rosetta^54^ taking into account the ring symmetry at all steps. The final model has a good fit to the map (Chimera correlation coefficient (CC) score 0.88) and good statistics (Molprobity^55^ score 1.92, Ramachandran favored 96.82% and outliers 0%, EMRinger^56^ score of 2.63). For InvG, the structure of the isolated closed conformation (PDB 5TCQ) was docked into the reconstruction using Chimera and the structurally different regions were rebuilt using COOT^57^. Both Phenix and Rosetta were used for refinement as above. The final model has good fit to the map (Chimera CC 0.87) and good statistics (Molprobity score 2.4, Ramachandran favored 94.76%, outliers 0%, EMRinger score of 1.23). For the isolated needle, the density permitted de novo model building in COOT^57^ and further refinement in both Phenix and Rosetta (using helical symmetry restraints). The final model has a good fit to the map (Chimera CC 0.92) and good statistics (Molprobity score 2.7, Ramachandran favored 96.05%, outliers 0%) and an EMRinger score of 1.83.

### Liquid chromatography tandem mass spectrometry

In-gel digestion for the basal body sample was carried out as described^58^. Digested proteins were loaded onto a Bruker Impact II Q-ToF mass spectrometer. Peptide separation was carried out on a 50 cm in-house packed 75 μm C18 column by a Proxeon EasynLC UPLC system, using 120 min water:acetonitrile gradients. Eluted peptides were ionized in positive ion mode, collecting MS/MS spectra for the top 15 peaks >500 counts, with a 30 sec dynamic exclusion list.

Resulting data files were loaded into MaxQuant v1.5.1.0 for analysis^59^. Identification used a 0.006 da MS tolerance and 40 ppm MSMS tolerance, and a *Salmonella* specific protein database containing all annotated *Salmonella* proteins extracted from Uniprot.

## Electronic supplementary material

Supplementary Information

## Data Availability

Data supporting the findings of this manuscript are available from the corresponding author upon reasonable request. Cryo-EM maps and atomic coordinates have been deposited with the EMDB and PDB with accession codes EMD-8913, EMD-8914, EMD-8915, EMD-8924, PDB ID 6DUZ, 6DV3, 6DV6, and 6DWB. The mass spectrometry data are available via ProteomeXchange with identifier PXD010239.
