# Supplementary material for: Cryo-EM analysis of the T3S injectisome reveals the structure of the needle and open secretin
Source: Nat Commun. 2018 Sep 21;9:3840. doi: 10.1038/s41467-018-06298-8 (PMC6155069; doi:10.1038/s41467-018-06298-8)
Supplement: Supplementary file 1 — Supplementary Information [file 41467_2018_6298_MOESM1_ESM.pdf]

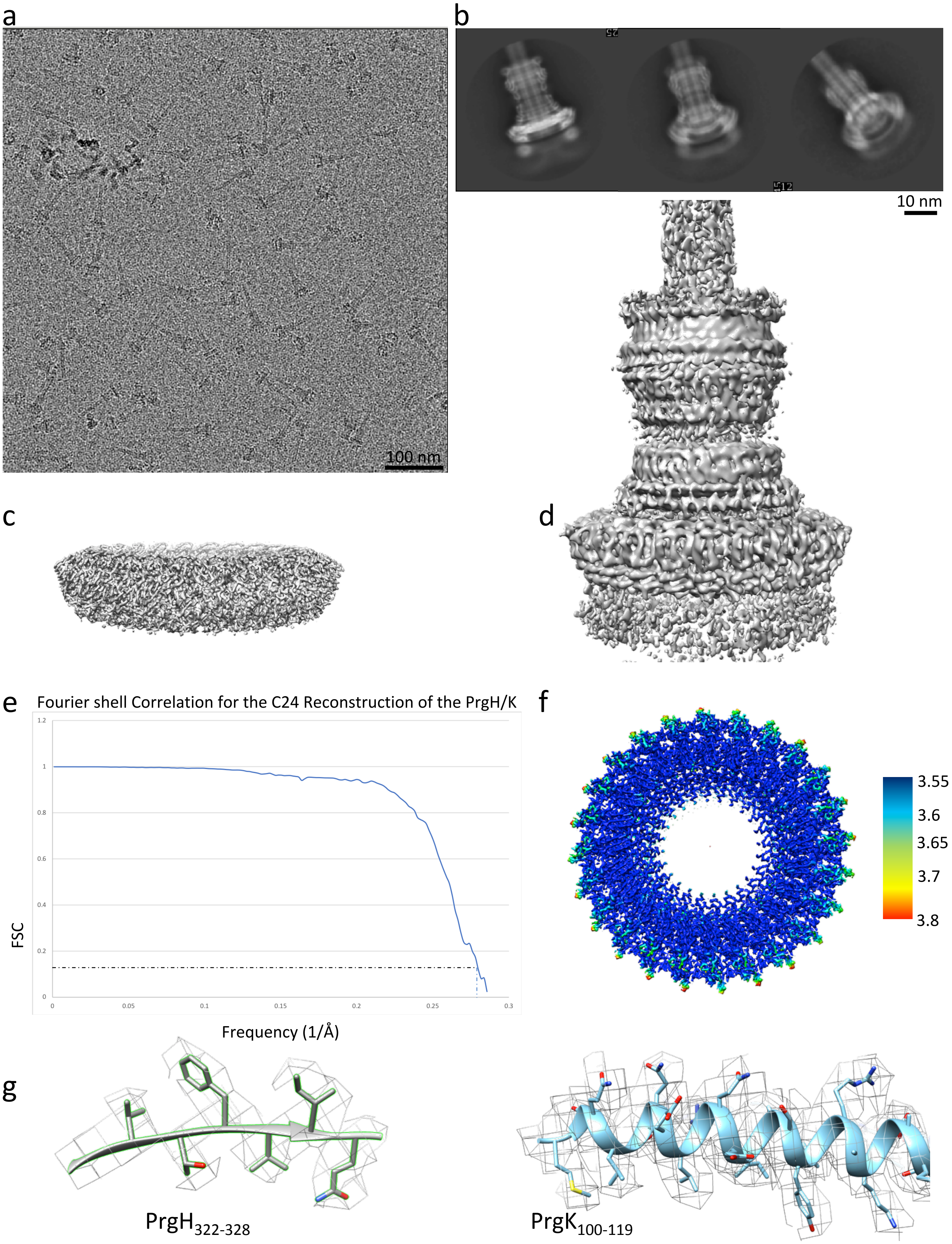

a
100 nm
b
10 nm
c
d
e
Fourier shell Correlation for the C24 Reconstruction of the PrgH/K
FSC
1.2
1
0.8
0.6
0.4
0.2
0
0
0.05
0.1
0.15
0.2
0.25
0.3
Frequency (1/Å)
f
3.55
3.6
3.65
3.7
3.8
g
PrgH322-328
PrgK100-119

Supplementary Fig. 1: 3D reconstructions of the needle complex and 24-fold symmetry averaged inner membrane rings. (a) Representative micrograph of the needle complex. **(b)** Selected reference-free 2D class averages. **(c)** C24 symmetry averaged inner membrane region at 3.6 Å resolution **(d)** C1 (no symmetry imposed) reconstructed map of the needle complex at 7.4 Å resolution. **(e)** FSC of the C24 reconstruction calculated in Relion. **(f)** Local resolution estimations of the C24 map from ResMap. Bottom slabbed view of the C24 reconstructed map. **(g)** Representative density for the inner membrane rings (3.6 Å resolution).

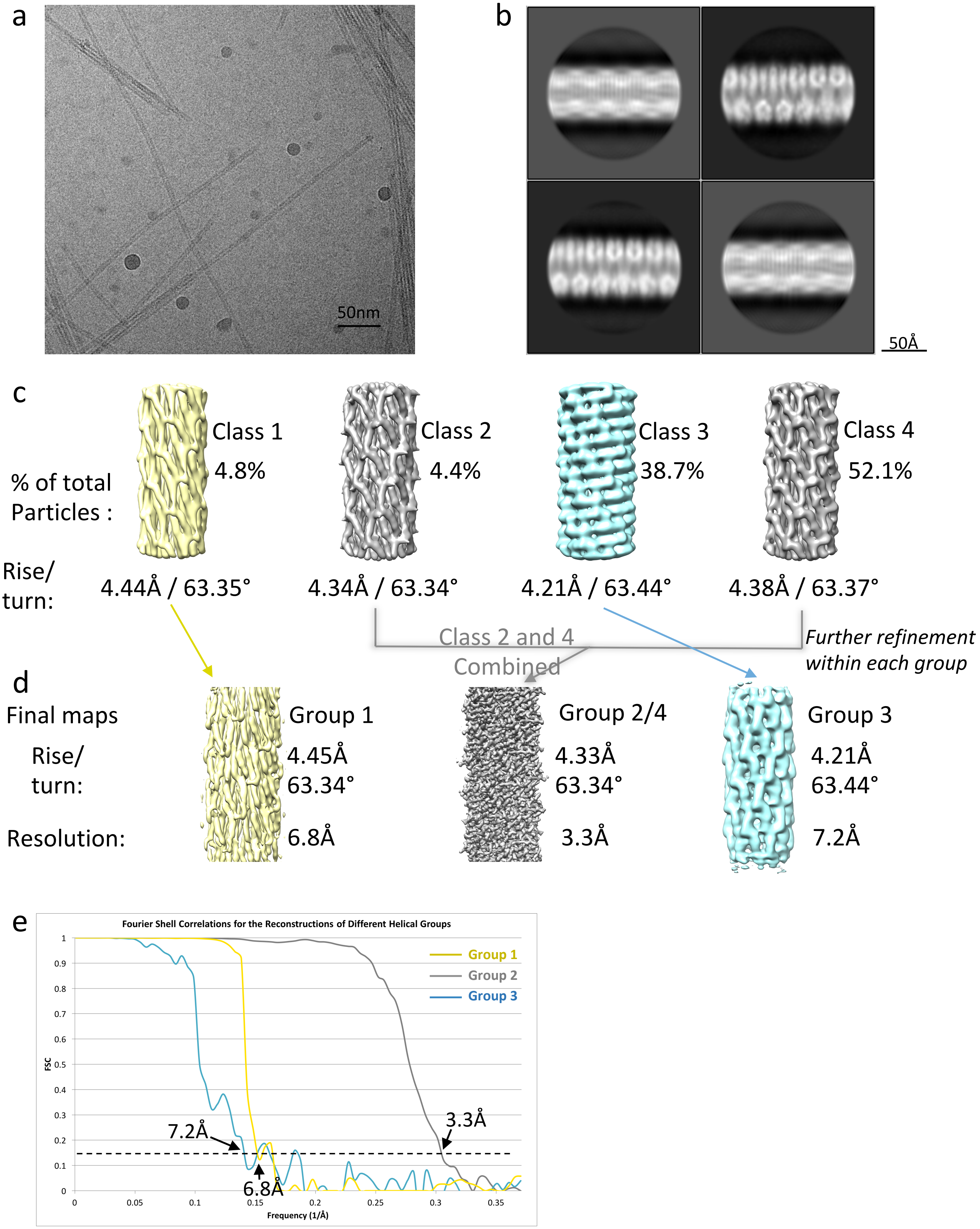
a
50nm
b
50Å
c
Class 1
4.8%
Class 2
4.4%
Class 3
38.7%
Class 4
52.1%
% of total Particles :
Rise/ turn:
4.44Å / 63.35°
4.34Å / 63.34°
4.21Å / 63.44°
4.38Å / 63.37°
Class 2 and 4 Combined
Further refinement within each group
d
Final maps
Rise/ turn:
Resolution:
Group 1
4.45Å
63.34°
6.8Å
Group 2/4
4.33Å
63.34°
3.3Å
Group 3
4.21Å
63.44°
7.2Å
e
Fourier Shell Correlations for the Reconstructions of Different Helical Groups
Group 1
Group 2
Group 3
FSC
Frequency (1/Å)
7.2Å
6.8Å
3.3Å

Supplementary Fig. 2: 3D reconstruction of the isolated needle at 3.3 Å resolution. (a) Representative micrograph of the isolated needle. **(b)** Selected reference-free 2D class averages. **(c)** 3D classes, class 2 and 4 combined for final high-resolution refinement. **(d)** Final maps for different classes revealing variation in helical rise. Class 2/4 refined to high resolution. **(e)** FSC of the different helical reconstructions.

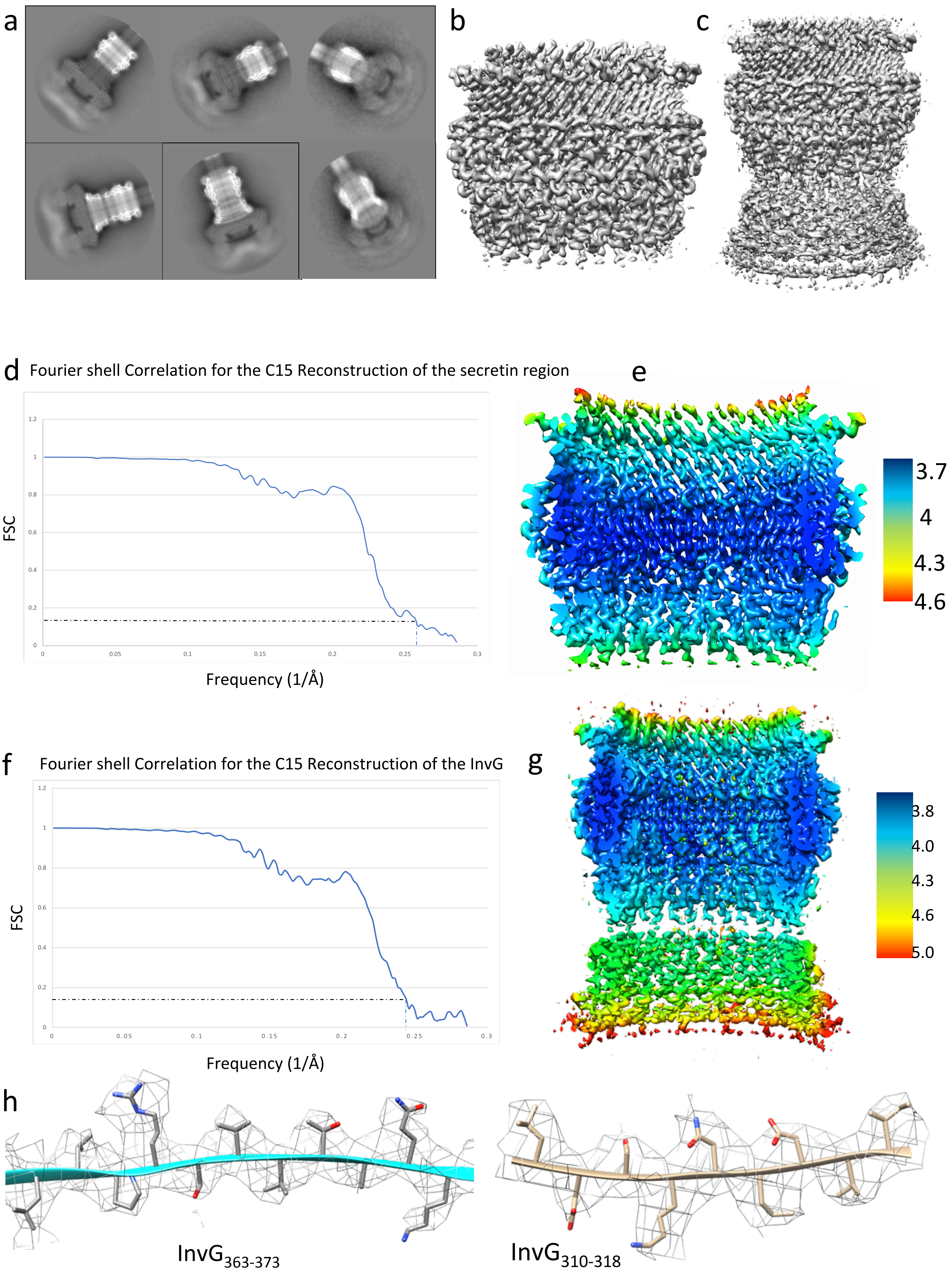
a
b
c
d
Fourier shell Correlation for the C15 Reconstruction of the secretin region
FSC
Frequency (1/Å)
e
3.7
4
4.3
4.6
f
Fourier shell Correlation for the C15 Reconstruction of the InvG
FSC
Frequency (1/Å)
g
3.8
4.0
4.3
4.6
5.0
h
InvG363-373
InvG310-318

Supplementary Fig. 3: 3D reconstructions of the 15-fold symmetry averaged OM secretin. (a) Selected local reference-free 2D class averages of the core N3, secretin and S domains (top row) or full-length secretin (bottom row). **(b)** C15 symmetry averaged core secretin at 3.9 Å resolution **(c)** C15 symmetry averaged full-length secretin at 4.1 Å resolution. **(d)** FSC for core secretin. **(e)** Local resolution estimations of core secretin from ResMap. Slabbed view. **f)** FSC for full-length secretin. **(g)** Local resolution estimations of full-length secretin from ResMap. Slabbed view. **(h)** Representative density for the core secretin (3.9 Å resolution).

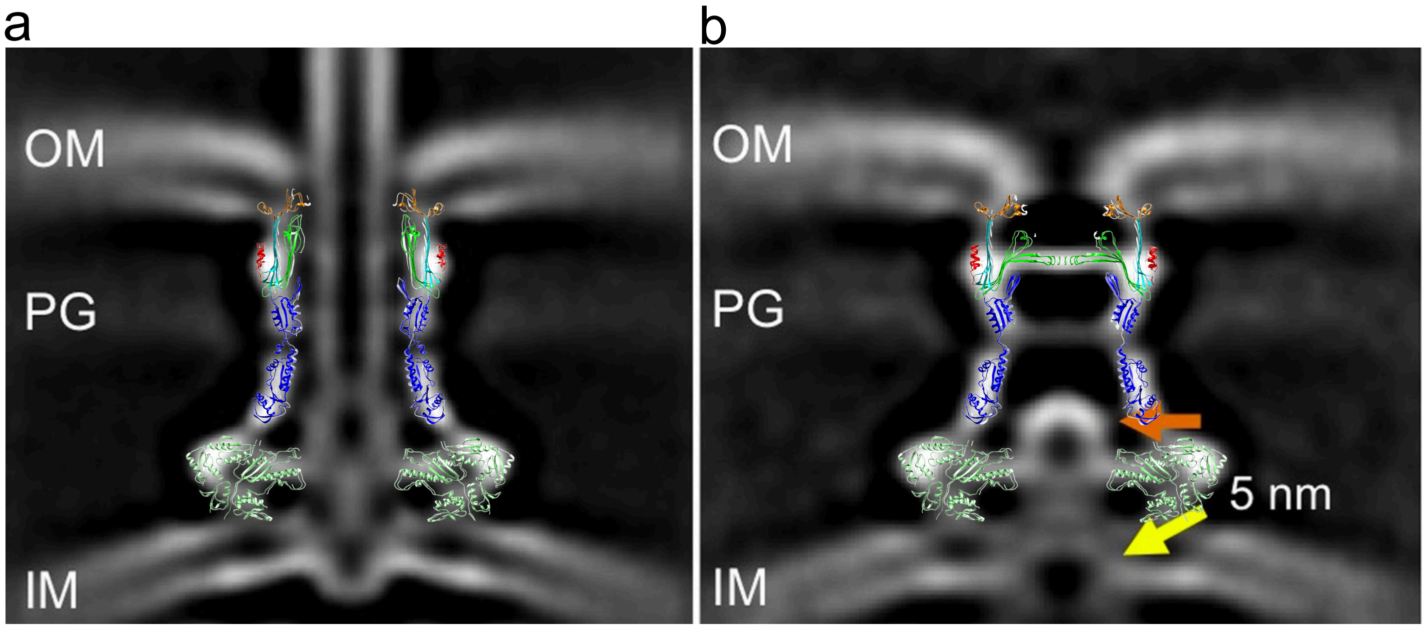

Supplementary Fig. 4: Comparison of isolated and *in situ* needle complex and basal body. Overlay of needle complex **(a)** and basal body **(b)** atomic models on the *in situ* cryo-ET image of the *S.* Typhimurium T3SS (figure reproduced from[13]). The relative structural span is conserved suggesting extraction from the membrane does not alter the overall structure significantly. Notably, the degree of membrane invagination caused by insertion of the needle is correlated to orientation of the secretin lip β-barrel. Reprinted from reference 13, with permission from Elsevier.

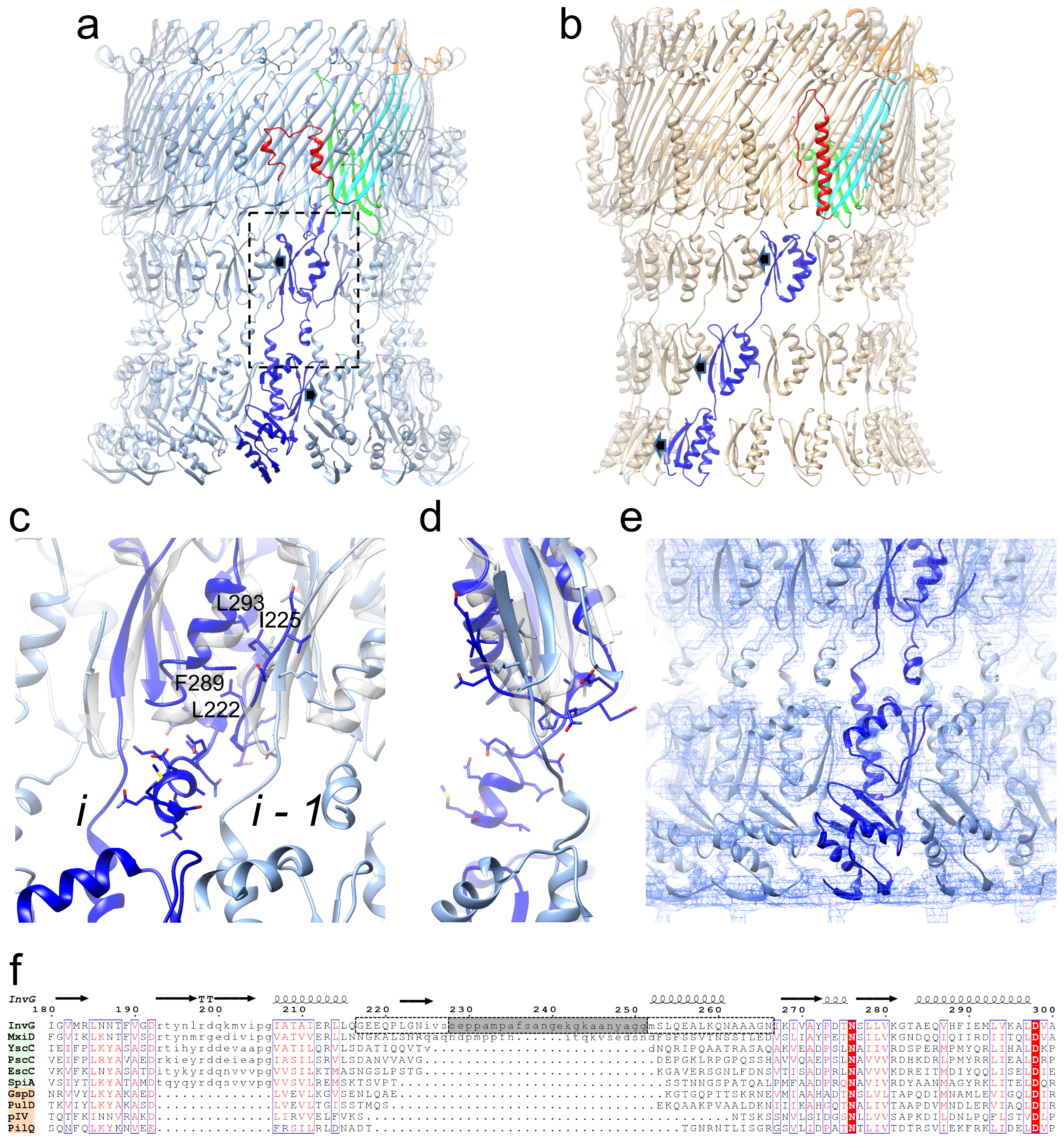

Supplementary Fig. 5: Packing of the peripheral N-terminal domains. (a) and **(b)** Secretin structures from the needle complex here and the T2SS secretin GspD (PDB 5WQ8). One monomer colored according to Fig. 1. Arrows indicate direction of RBM interface. **(c)** and **(d)** Zoomed view of N3 domain (boxed in a) showing packing of ordered regions of N3 domain loop (shown as sticks). The isolated secretin (closed state) structure is overlaid in transparent grey illustrating the packing of the N-terminal most ordered region, which is folded back against the N3 domain. **(e)** Density for the N0, N1 and N3 domains, the N1 and N0 domains are less well resolved (also see Supplementary Fig. 3g) although clear secondary structure density allowed positioning of the InvG$_{33\text{-}172}$ crystal structure and further refinement. **(f)** Multiple sequence alignment for the N3 domain from T3SS (green) and non-T3SS (orange) secretins. The variable N3 domain loop further resolved here is boxed with the region still disordered shaded grey.

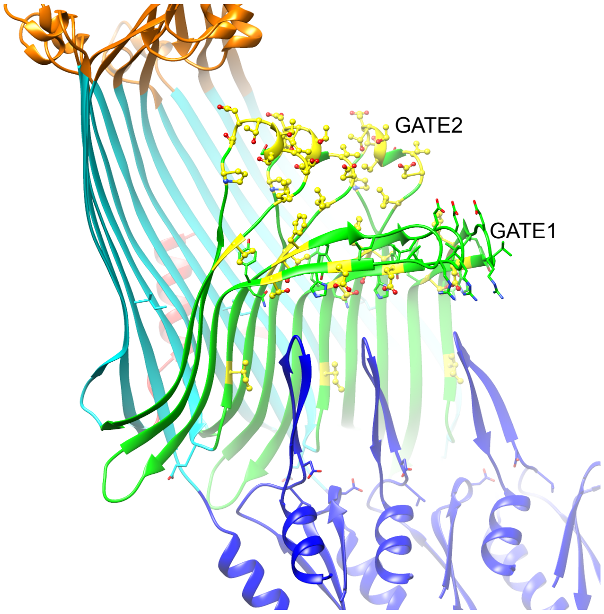

Supplementary Fig. 6: Mutations found to affect filamentous phage secretin pIV permeability[35] mapped onto the InvG structure. Mutants map to the N3-secretin interface, the outer β-barrel wall and mostly to the GATE1 and GATE2 hairpins (shown as stick or ball and stick). The mutants found to increase sensitivity to antibiotics, suggesting a bigger impact on the degree of gate opening, are coloured yellow and show as ball and stick with a high concentration mapping to GATE2.

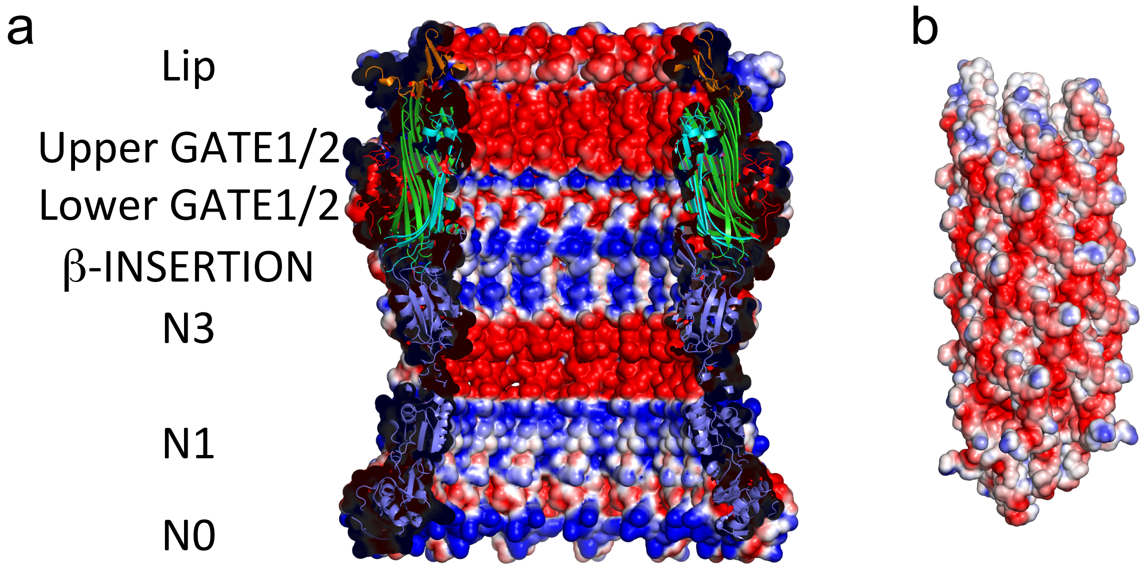

Supplementary Fig. 7: Electrostatic surface for InvG in the open state and the PrgI needle. Surface electrostatics calculated with APBS[60] for the interior of the open InvG pore **(a)** and the PrgI needle **(b)**. The InvG N1 and upper N3 domain including the β-INSERTION are mainly positively charged while the lower N3 domain and upper regions of the extended GATE1 and GATE hairpins and lip are electronegative. The β-INSERTION packs closely with the assembled needle filament within the secretin lumen and the charge complementarity with the predominantly negatively charged needle exterior suggest a possible means of further anchoring the needle within the basal body.

	#1 Needle complex C1 map	#2 InvG$_{34-557}$ C15 map (EMDB-8915) (PDB 6DV6)	#3 InvG$_{176-557}$ C15 map (EMDB-8914) (PDB 6DV3)	#4 PrgH/K ring C24 map (EMDB-8913) (PDB 6DUZ)	#5 PrgI Needle Filaments (EMDB-8924) (PDB 6DWB)
**Data collection and processing**					
Magnification	47000	47000	47000	47000	37037
Voltage (kV)	300	300	300	300	300
Electron exposure (e–/Å^2)	40	40	40	40	55
Defocus range (μm)	1.25-4	1.25-4	1.25-4	1.25-4	1-2.5
Pixel size (Å)	1.75	1.75	1.75	1.75	0.625
Symmetry imposed	C1	C15	C15	C24	helical
Initial particle images (no.)	80000	80000	80000	80000	867000
Final particle images (no.)	42647	26280	26000	58198	53000
Map resolution (Å)<br>FSC threshold	0.143	0.143	0.143	0.143	0.143
**Refinement**					
Initial model used (PDB code)		5TCQ	5TCQ	5TCP	
Model resolution range (Å)	7.4	4.1	3.9	3.6	3.3
Map sharpening *B* factor (Å^2)	-178	-141	-144	-118	-145
Model composition<br>Non-hydrogen atoms<br>Protein residues<br>Ligands		 57975<br>7500	 40950<br>5372	 72768<br>9072	 18360<br>2340
B factors (Å^2)<br>Protein<br>Ligand		9.9	13.87	11	19.6
R.m.s. deviations					
Bond lengths (Å)		0.009	0.010	0.007	0.016
Bond angles (°)		1.37	1.49	1.25	1.3
Validation					
MolProbity score		2.4	2.54	1.92	2.7
Clashscore		8.63	8.13	4.63	6.12
Poor rotamers (%)		8.59	7.64	4.3	0
Ramachandran plot					
Favored (%)		5.24	6.5	3.18	3.95
Allowed (%)		94.76	93.5	96.82	96.05
Disallowed (%)		0	0	0	0

Supplementary Table 1. Cryo-EM data collection, refinement and validation statistics.

Protein ID	MW (kDa)	Peptides	Relative intensity
PRGK_SALTY	30	32	1
PRGI_SALTY	8.9	16	0.92
PRGH_SALTY	44.5	48	0.74
INVG_SALTY	61.7	64	0.30
PRGJ_SALTY	10.9	10	0.16
SPAP_SALTY	25.2	9	0.06
SIPD_SALTY	37.1	19	0.01
SIPA_SALTY	73.9	18	0.005
HILA_SALTY	63	8	0.002
INVA_SALTY	76.1	10	0.002
SPAR_SALTY	28.5	1	0.001
INVH_SALTY	16.5	2	0.0004
SIPB_SALTY	62.5	2	0.0003

Supplementary Table 2. Mass spectrometry analysis of the purified needle complex.
